# Socioeconomic deprivation, urban-rural location and alcohol-related mortality in England and Wales

**DOI:** 10.1186/1471-2458-10-99

**Published:** 2010-02-25

**Authors:** Sally Erskine, Ravi Maheswaran, Tim Pearson, Dermot Gleeson

**Affiliations:** 1 Public Health GIS Unit, School of Health and Related Research, University of Sheffield, Regent Court, 30 Regent Street, Sheffield, S1 4DA, UK; 2 Liver Unit, Royal Hallamshire Hospital, Glossop Road, Sheffield, S10 2JF, UK

## Abstract

**Background:**

Many causes of death are directly attributable to the toxic effects of alcohol and deaths from these causes are increasing in the United Kingdom. The aim of this study was to investigate variation in alcohol-related mortality in relation to socioeconomic deprivation, urban-rural location and age within a national context.

**Methods:**

An ecological study design was used with data from 8797 standard table wards in England and Wales. The methodology included using the Carstairs Index as a measure of socioeconomic deprivation at the small-area level and the national harmonised classification system for urban and rural areas in England and Wales. Alcohol-related mortality was defined using the National Statistics definition, devised for tracking national trends in alcohol-related deaths. Deaths from liver cirrhosis accounted for 85% of all deaths included in this definition. Deaths from 1999-2003 were examined and 2001 census ward population estimates were used as the denominators.

**Results:**

The analysis was based on 28,839 deaths. Alcohol-related mortality rates were higher in men and increased with increasing age, generally reaching peak levels in middle-aged adults. The 45-64 year age group contained a quarter of the total population but accounted for half of all alcohol-related deaths. There was a clear association between alcohol-related mortality and socioeconomic deprivation, with progressively higher rates in more deprived areas. The strength of the association varied with age. Greatest relative inequalities were seen amongst people aged 25-44 years, with relative risks of 4.73 (95% CI 4.00 to 5.59) and 4.24 (95% CI 3.50 to 5.13) for men and women respectively in the most relative to the least deprived quintiles. People living in urban areas experienced higher alcohol-related mortality relative to those living in rural areas, with differences remaining after adjustment for socioeconomic deprivation. Adjusted relative risks for urban relative to rural areas were 1.35 (95% CI 1.20 to 1.52) and 1.13 (95% CI 1.01 to 1.25) for men and women respectively.

**Conclusions:**

Large inequalities in alcohol-related mortality exist between sub-groups of the population in England and Wales. These should be considered when designing public health policies to reduce alcohol-related harm.

## Background

Many causes of death are directly attributable to the toxic effects of alcohol. Such deaths include those from alcoholic liver cirrhosis, alcoholic pancreatitis, alcoholic gastritis, alcohol poisoning and alcoholic cardiomyopathy. Deaths from these disorders are increasing in the United Kingdom whilst they are declining in several other countries in Europe [1,2]. Despite attempts to inform the public about safe levels of alcohol consumption, alcohol continues to be used harmfully [3]. 38% of men and 16% of women aged 16 to 64 years have an alcohol use disorder, which is equivalent to approximately 8.2 million people in England [4]. Binge drinking is increasingly common and is particularly associated with harmful consequences, including increased all cause mortality and mortality from directly alcohol-related causes [5].

Several national reports have highlighted the above issues and suggested possible ways to reduce alcohol-related harm [6-8]. Amongst these suggestions, the possibility of focussing strategies towards the minority of drinkers who experience the most harm related to alcohol has been proposed. Deaths from alcohol-related causes represent one extreme of the physical harm caused by alcohol. Identification of individuals who are most likely to die from an alcohol-related cause may therefore be useful in the design of effective alcohol harm reduction policies and in the allocation of resources to reduce alcohol-related harm, and may provide additional information about the association between alcohol consumption and the risk of dying from an alcohol-related disorder.

It is thought that certain sub-groups of the population may experience greater levels of alcohol-related mortality, including younger age groups and socioeconomically disadvantaged individuals [9,10]. A recent study in England and Wales reported higher levels of alcohol-related mortality in more socioeconomically deprived areas but did not examine the age groups in which the adverse socioeconomic effect was most clearly seen [11]. Urbanicity was also not taken into account and some previous studies [12,13] but not others [14] have found higher alcohol-related mortality rates in urban areas.

The aim of our study was to investigate variation in alcohol-related mortality in relation to socioeconomic deprivation, urban-rural location and age within a national context.

## Methods

We used an ecological (geographical) study design for our investigation. Data were available for standard table wards in England and Wales. These are similar to electoral wards and are a standard geographical unit for which 2001 Census information is available.

We used the National Statistics definition of alcohol-related mortality which had been agreed by the Office for National Statistics, the General Register Office for Scotland and the Northern Ireland Statistics and Research Agency, for tracking national trends in alcohol-related deaths [11,15]. The definition only included causes of death regarded as being most directly attributable to alcohol consumption. It included all deaths from chronic liver disease and cirrhosis (excluding biliary cirrhosis) and these deaths accounted for 85% of all alcohol-related deaths included in the definition. Other diseases in the definition included mental, behavioural and nervous system disorders and degeneration due to alcohol, alcohol poisoning, alcohol-induced pancreatitis and alcoholic cardiomyopathy.

We used a dataset which had been assembled by the Office for National Statistics for surveillance of alcohol-related mortality [11]. It included all standard table wards in England and Wales. The dataset supplied comprised 8797 wards (one of which was an amalgamation of four City of London wards) and contained deaths from 1999-2003 by five-year age band and sex, allocated to wards based on the deceased's usual place of residence. The corresponding denominator population counts supplied with the dataset were from the 2001 census ward population estimates. The dataset included Carstairs Index as a measure of socioeconomic deprivation at the ward level [16]. It is a standardised combination of four 2001 Census ward-level variables: the proportion of economically active men who were unemployed; the proportion of individuals living in overcrowded accommodation; the proportion of the population where the head of the household was from Social Class IV or V; and the proportion of residents in households with no car. Wards were categorised into quintiles based on their deprivation score, with each quintile containing a fifth of the population of England and Wales.

To investigate urbanicity, we used the national harmonised classification system for urban and rural areas in England and Wales developed by the Countryside Agency, the Office for National Statistics, the Department for Environment, Food and Rural Affairs, the Department for Communities and Local Government and the National Assembly for Wales [17]. Each ward was classified in terms of settlement type. The categories were: urban; town and fringe; and village, hamlet and isolated dwelling.

We calculated age-specific mortality rates to examine the effects of socioeconomic deprivation graphically. We used Poisson regression (Genmod procedure in SAS version 9.1) to carry out statistical analyses. Poisson regression is appropriate for modelling counts and assumes that they follow a Poisson distribution. Data were grouped by sex, five-year age band, deprivation quintile and urban-rural status before being entered into regression models. Population denominators were used as the offset and models were adjusted for overdispersion. The results are presented as adjusted rate ratios with 95% confidence intervals (CI).

## Results

The analysis was based on 18,716 deaths in males and 10,123 deaths in females from 1999 to 2003 (table 1). The denominator population was 25,574,258 males and 26,785,720 females, giving an overall annual alcohol-related mortality rate of 14.6 per 100,000 population for males and 7.6 per 100,000 population for females. The 45-64 year age group contained a quarter of the total population but accounted for half of all alcohol-related deaths (table 1).

**Table 1:** Distribution of alcohol-related deaths (1999-2003) and population (2001) by age, sex, socioeconomic deprivation and urban-rural morphology in England and Wales.

	Alcohol-related deaths	Proportion of alcohol-related deaths (%)	Population	Proportion of population (%)
**Age (years)**				
Males				
<15	4	0.02	5035663	19.69
15-24	81	0.43	3231330	12.64
25-44	3727	19.91	7638862	29.87
45-64	10228	54.65	6158513	24.08
65-79	3916	20.92	2785671	10.89
80+	760	4.06	724219	2.83
Total	18716	100.00	25574258	100.00
				
Females				
<15	2	0.02	4795708	17.90
15-24	22	0.22	3156103	11.78
25-44	1880	18.57	7713420	28.80
45-64	5019	49.58	6288812	23.48
65-79	2493	24.63	3346180	12.49
80+	707	6.98	1485497	5.55
Total	10123	100.00	26785720	100.00
				
**Socioeconomic deprivation quintile**				
Males				
1 - least deprived	2082	11.12	5131382	20.06
2	2719	14.53	5104533	19.96
3	3510	18.75	5092958	19.91
4	4478	23.93	5102748	19.95
5 - most deprived	5927	31.67	5142637	20.11
Total	18716	100.00	25574258	100.00
				
Females				
1 - least deprived	1404	13.87	5334961	19.92
2	1677	16.57	5366494	20.03
3	1993	19.69	5384588	20.10
4	2409	23.80	5368947	20.04
5 - most deprived	2640	26.08	5330730	19.90
Total	10123	100.00	26785720	100.00
				
**Urban-rural morphology**				
Males				
Village, hamlet and isolated dwelling	958	5.12	2371452	9.27
Town and fringe	1497	8.00	2611270	10.21
Urban	16261	86.88	20591536	80.52
Total	18716	100.00	25574258	100.00
				
Females				
Village, hamlet and isolated dwelling	673	6.65	2413067	9.01
Town and fringe	929	9.18	2738839	10.22
Urban	8521	84.17	21633814	80.77
Total	10123	100.00	26785720	100.00

Urban areas accounted for approximately 80% of the total population and 85% of all alcohol-related deaths while villages accounted for approximately 9% of the population and 6% of alcohol-related deaths. The most socioeconomically deprived 20% of the population of England and Wales accounted for 32% of alcohol-related deaths in men and 26% of alcohol-related deaths in women whilst the least deprived 20% of the population accounted for 11% of male and 14% of female alcohol-related deaths (table 1).

**Figure 1:**
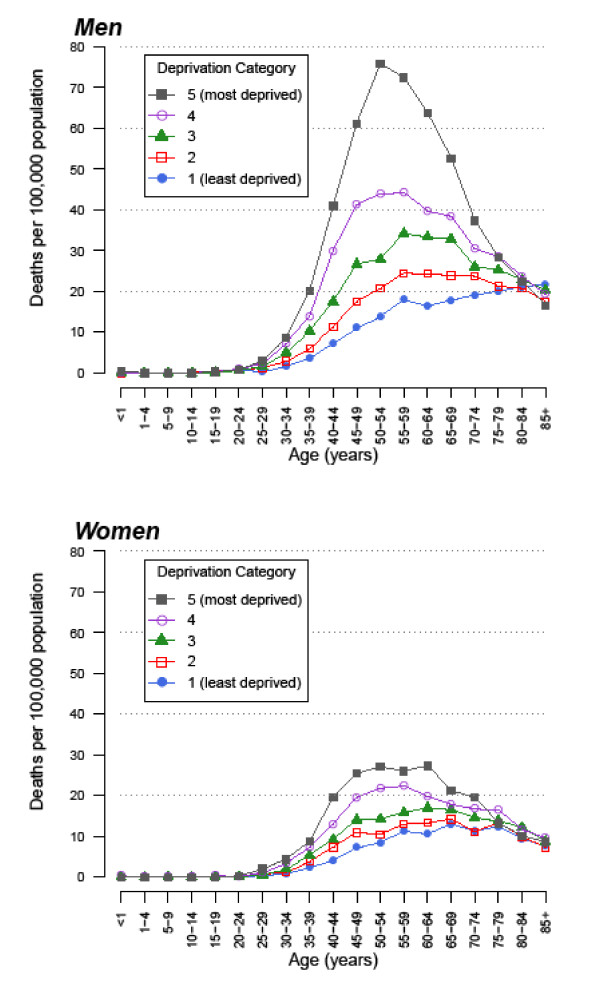
**Annual alcohol-related mortality rates in England and Wales based on deaths from 1999-2003 by sex, age and socioeconomic deprivation quintile (1 = least deprived, 5 = most deprived)**.

Figure 1 shows age-specific rates for alcohol-related mortality by gender and socioeconomic deprivation. Rates were higher in men and increased with increasing age, generally reaching peak levels in middle-aged adults before declining in older adults. The figure clearly demonstrates the association between alcohol-related mortality and socioeconomic deprivation, with progressively higher rates in more deprived categories. The overall pattern suggests that the peak occurred at a slightly younger age with increasing socioeconomic deprivation.

Table 2 shows rate ratios (relative risks) for alcohol-related mortality adjusted for age and urban-rural status for the most, compared with the least, socioeconomically deprived fifth of the population in England and Wales. For all ages combined, the adjusted relative risks were 3.39 (95% CI 3.10 to 3.71) in men and 2.36 (95% CI 2.16 to 2.57) in women. The strength of the association, however, varied with age. The greatest inequalities were seen amongst people aged 25-44 years, with relative risks of 4.73 (95% CI 4.00 to 5.59) and 4.24 (95% CI 3.50 to 5.13) for men and women respectively in the most relative to the least deprived categories.

**Table 2:** Alcohol-related mortality rate ratios by age category, for the most, relative to the least, socioeconomically deprived quintile of the population of England and Wales, 1999-2003.

	Rate ratio (95% CI)	
Age	Males	Females
15-24 years	1.45 (0.65 to 3.27)	Too few deaths
25-44 years	4.73 (4.00 to 5.59)	4.24 (3.50 to 5.13)
45-64 years	4.18 (3.76 to 4.65)	2.67 (2.40 to 2.96)
65-79 years	2.01 (1.75 to 2.32)	1.48 (1.29 to 1.71)
80+ years	0.97 (0.75 to 1.26)	1.13 (0.86 to 1.48)
		
All ages	3.39 (3.10 to 3.71)	2.36 (2.16 to 2.57)

Rate ratios (95% confidence intervals) have been adjusted for urban-rural status and five-year age band within the broader age categories shown.

Table 3 shows alcohol-related mortality rate ratios for urban-rural categories adjusted for age, before and after adjustment for socioeconomic deprivation. People living in urban areas experienced higher alcohol-related mortality relative to those living in rural areas, with differences remaining after additional adjustment for socioeconomic deprivation. Adjusted relative risks for urban relative to rural areas were 1.35 (95% CI 1.20 to 1.52) and 1.13 (95% CI 1.01 to 1.25) for men and women respectively.

**Table 3:** Association between alcohol-related mortality and urbanicity in England and Wales, 1999-2003.

	Rate ratio (95% CI)	
Urbanicity	Adjusted for age	Adjusted for age and socioeconomic deprivation
**Males**		
Village, hamlet and isolated dwelling	1	1
Town and fringe	1.51 (1.26 to 1.82)	1.19 (1.04 to 1.37)
Urban	2.37 (2.05 to 2.75)	1.35 (1.20 to 1.52)
		
**Females**		
Village, hamlet and isolated dwelling	1	1
Town and fringe	1.28 (1.10 to 1.48)	1.08 (0.95 to 1.22)
Urban	1.68 (1.50 to 1.88)	1.13 (1.01 to 1.25)

Rate ratios (95% confidence intervals) adjusted for age, before and after adjustment for socioeconomic deprivation.

## Discussion

We found that alcohol-related mortality rates were higher in men and generally reached peak levels amongst middle-aged adults. The 45-64 year age group contained a quarter of the total population of England and Wales but accounted for half of all alcohol-related deaths. There was a clear increase in alcohol-related mortality with increasing socioeconomic deprivation. The strength of the association varied with age and the greatest relative inequalities were seen amongst people aged 25-44 years, with mortality rates for men and women in the most deprived quintile over four times the rates in the least deprived quintile. Rates were also higher in urban areas, even after adjustment for socioeconomic deprivation.

The observation that alcohol-related mortality increases with increasing socioeconomic deprivation is supported by other studies [12,18]. Differences in alcohol-related mortality between different socioeconomic groups have often been found to be greater amongst men [9,13,18] although some studies have observed greater differences amongst women [19,20]. Some studies have also found greater socioeconomic differences in alcohol-related mortality rates amongst younger people, consistent with our findings [9,10,12]. A number of these studies were carried out in different countries including Finland, the USA and Russia.

We found that alcohol-related mortality was higher in urban areas. Studies in different populations have also observed a greater risk of dying from an alcohol-related cause in urban areas [13,18]. A study investigating alcohol-related mortality in relation to geodemographic clusters found mortality amongst wealthy older people in rural areas to be relatively low [3]. In contrast, lower alcohol-related mortality in urban areas, after adjustment for individual level characteristics, has also been reported [14].

Our results, which show substantially elevated alcohol-related mortality in relation to socioeconomic deprivation, appear to be at odds with national survey data on alcohol consumption patterns [21,22]. The General Household Survey, a multi-purpose continuous survey started in 1971 and currently carried out by the Office for National Statistics, has consistently found no excess alcohol consumption in the more socioeconomically deprived groups [21]. In the 2006 survey, men in the "routine and manual" group were drinking on average 16.7 units a week, considerably less than the 19.9 units for men in the "managerial and professional" and "intermediate" groups. Amongst women, average weekly consumption was highest, at 10.7 units, in the managerial and professional group, and lowest, at 7.1 units, among those in routine and manual worker households. With regard to binge drinking, 24% of men in the managerial and professional group reported drinking more than 8 units on at least one day in the previous week, compared with 21% in the manual group. The corresponding figures for women were 17% and 12% drinking more than 6 units on at least one day in the previous week. These results are supported by data from the Health Survey for England, which show that higher income groups consumed alcohol more frequently, and had a higher prevalence of binge drinking than people in lower income groups [22]. These findings are surprising, given the strong adverse socioeconomic effects we have observed in relation to alcohol-related mortality. It is possible that selection bias in the samples obtained, or information bias in the recall of alcohol consumption, could have resulted in underestimation of alcohol intake in the lower socioeconomic groups, although this would call into question the validity of alcohol data from these national surveys. Variations in the extent to which the area-level deprivation index and employment groups correspond to socioeconomic deprivation may contribute to the apparent discrepancy. Ecological bias could also contribute to the apparent discordant patterns. In contrast to the survey results, analysis of data from the 1958 British birth cohort study showed that whilst less educated men and women had greater odds of being non-drinkers, they also had greater odds of binge drinking [23].

The discrepancy between our study results based on mortality data and the results regarding drinking behaviour from national studies described above might also imply that socioeconomically deprived heavy drinkers are more likely to get serious liver disease than affluent heavy drinkers. Susceptibility to alcoholic liver disease is very variable and one possible reason may be obesity which is a risk factor for severe alcoholic liver disease [24]. Both the type of alcohol consumed and whether or not food is consumed at the same time may affect subsequent risk of alcohol-related mortality [25]. Consumption of different types of fats and overall nutritional status may also influence the risk of developing an alcohol-related illness [26]. Patterns of alcohol consumption, diet and obesity could vary with socioeconomic status and potentially explain the apparent discordant patterns in relation to alcohol consumption and alcohol-related mortality.

The higher mortality amongst those experiencing greater levels of deprivation could be due to higher incidence of alcohol-related disorders or to poorer survival following development of alcohol-related disorders, or both. The level of support received for management of alcohol-related problems may vary with socioeconomic status. Inequalities in access to healthcare interventions are known to exist for many illnesses, with more socioeconomically deprived individuals receiving fewer beneficial interventions. The need for further investigation into such inequalities in relation to alcohol-related disorders has been highlighted [4]. However, a study in Finland found that socioeconomic differences in alcohol-related hospital admission rates were almost as large as those observed for alcohol-related mortality. Survival after discharge was found to be similar among different socioeconomic groups or worse amongst the less deprived groups depending on the age category considered [27]. This suggests that the higher alcohol-related mortality in more deprived groups may be due to higher incidence of alcohol-related morbidity rather than poorer survival.

The association between area-level deprivation and alcohol-related mortality could be explained by social drift, that is the tendency for individuals with hazardous alcohol consumption to move to live in deprived areas. In addition, genetic differences between individuals experiencing different levels of deprivation have been proposed in relation to alcohol-related disorders, as the social drift down a socioeconomic hierarchy due to alcohol consumption could lead to genetic drift whereby those with genetic predisposition to alcoholism or alcohol-related harm are over-represented in more socioeconomically deprived groups [18]. A combination of more harmful alcohol consumption, poorer general health and genetic predisposition to developing alcohol-related disease could therefore lead to more deprived individuals dying from alcohol-related causes at younger ages than those who are less deprived.

A number of potential limitations to our study need to be considered. All deaths from liver fibrosis and cirrhosis, other than those due to biliary cirrhosis, were included but not all of these deaths will have been caused by alcohol. A large proportion of non-alcohol related deaths from liver disease could introduce bias if the causes of these deaths are unevenly distributed, and higher proportions in more deprived areas could overestimate the observed association. The deaths included in the National Statistics definition of alcohol-related mortality are not the only causes of death which are attributable to alcohol consumption, but the definition is likely to have captured most liver-related deaths due to alcohol. The International Classification of Diseases 10^th ^Revision (ICD-10) was implemented in 2001 and could have resulted in inconsistencies in the classification of alcohol-related deaths over the study period. However, the Office for National Statistics carried out a bridging classification exercise to identify alcohol-related deaths occurring in 1999 using both ICD-9 and ICD-10 based definitions and found that inconsistencies were relatively minor [15].

Death certificates do not always accurately reflect an actual cause of death and the stigma attached to an alcohol-related cause may lead to under reporting of such deaths. The extent of under reporting may be influenced by characteristics of the individual, particularly age and socioeconomic status, potentially introducing bias into the associations observed. It is possible that fewer people in rural areas die in hospital and so are less likely to be labelled as having an alcohol-related death, resulting in bias in the urban-rural differences observed. Residual socioeconomic confounding could also have contributed to overestimation of the urban-rural differences. We used an ecological study design and the potential for ecological bias, where the association seen at the area level is different from that which exists at the individual level, cannot be ruled out. However, this may be less of a problem with small-area level ecological studies and wards are relatively small geographical areas within a national context. Issues related to inferential support and structural confounding due to selective migration also complicate interpretation of ecological study results [28].

## Conclusions

In summary, we found that whilst half of all alcohol-related deaths occurred in the 45-64 year age group, the greatest socioeconomic inequalities in relative terms were seen in the 25-44 year age group. We also found that people living in urban areas experienced higher risks of alcohol-related mortality after adjustment for socioeconomic deprivation. The large inequalities in alcohol-related mortality that exist between sub-groups of the population in England and Wales should be considered when designing public health policies to reduce alcohol-related harm.

## Competing interests

The authors declare that they have no competing interests.

## Authors' contributions

RM conceived the idea for the study. SE, TP and RM carried out the analyses. DG contributed to presentation, interpretation and discussion of the results. SE and RM drafted the manuscript with contributions from the other authors. All authors read and approved the final manuscript.

## Authors' information

SE, Medical student (Intercalated BMedSci).

RM, Clinical Senior Lecturer in Public Health Medicine.

TP, Research Associate in Public Health.

DG, Consultant Hepatologist.

## Pre-publication history

The pre-publication history for this paper can be accessed here:

http://www.biomedcentral.com/1471-2458/10/99/prepub
